# Predicting persistent inflammatory arthritis amongst early arthritis clinic patients in the UK: is musculoskeletal ultrasound required?

**DOI:** 10.1186/ar4298

**Published:** 2013-09-12

**Authors:** Arthur G Pratt, Alice R Lorenzi, Gill Wilson, Philip N Platt, John D Isaacs

**Affiliations:** 1 Musculoskeletal Research Group, Institute for Cellular Medicine, Newcastle University, Newcastle upon Tyne, UK; 2 Musculoskeletal Unit, The Freeman Hospital, Newcastle upon Tyne, UK; 3 Faculty of Health and Social Care, University of Hull, Hull, UK

## Abstract

**Introduction:**

Analyses of large clinical datasets from early arthritis cohorts permit the development of algorithms that may be used for outcome prediction in individual patients. The value added by routine use of musculoskeletal ultrasound (MSUS) in an early arthritis setting, as a component of such predictive algorithms, remains to be determined.

**Methods:**

The authors undertook a retrospective analysis of a large, true-to-life, observational inception cohort of early arthritis patients in Newcastle upon Tyne, UK, which included patients with inflammatory arthralgia but no clinically swollen joints. A pragmatic, 10-minute MSUS assessment protocol was developed, and applied to each of these patients at baseline. Logistic regression was used to develop two "risk metrics" that predicted the development of a persistent inflammatory arthritis (PIA), with or without the inclusion of MSUS parameters.

**Results:**

A total of 379 enrolled patients were assigned definitive diagnoses after ≥12 months follow-up (median 28 months), of whom 162 (42%) developed a persistent inflammatory arthritis. A risk metric derived from 12 baseline clinical and serological parameters alone had an excellent discriminatory utility with respect to an outcome of PIA (area under receiver operator characteristic (ROC) curve 0.91; 95% CI 0.88 to 0.94). The discriminatory utility of a similar metric, which incorporated MSUS parameters, was not significantly superior (area under ROC curve 0.91; 95% CI 0.89 to 0.94). Neither did this approach identify an added value of MSUS over the use of routine clinical parameters in an algorithm for discriminating PIA patients whose outcome diagnosis was rheumatoid arthritis (RA).

**Conclusions:**

MSUS use as a routine component of assessment in an early arthritis clinic did not add substantial discriminatory value to a risk metric for predicting PIA.

## Introduction

National and international guidelines increasingly emphasise the importance of early diagnosis in the management of new onset inflammatory arthritis and support the establishment of dedicated early arthritis (EA) clinics [1,2]. However, despite new classification criteria for rheumatoid arthritis (RA) [3,4], a substantial proportion of EA clinic attendees have un-classifiable disease, and are labelled as having undifferentiated arthritis (UA) [5,6].

Analyses of large clinical datasets from early arthritis cohorts permit the development of algorithms that may be used for outcome prediction in individuals - an approach which yielded a validated "prediction rule" for use in UA patients, in which a range of baseline clinical and laboratory parameters are weighted and combined to yield a score that relates to RA progression risk [7-9]. It has been suggested that every bit as clinically important as identifying individuals specifically destined for RA is the more general goal of predicting persistent inflammatory arthritis (PIA) amongst EA patients as a whole [10-12]. Hence, a predictive tool applicable to all EA clinic attendees, which distinguishes chronic inflammatory from non-inflammatory/self-limiting disease, could accelerate access to disease-modifying anti-rheumatic drugs (DMARDs) for those most likely to benefit from them. Musculoskeletal ultrasound has shown promise as an evaluation tool in the setting of early arthritis [13,14], but the value it adds to a thorough clinical assessment, for example, as a component of a predictive algorithm, remains to be quantified.

The Newcastle EA Clinic accepts patients clinically suspected of having new-onset inflammatory arthritis by their referring physician [15]. Blood test results and/or the presence of clinically inflamed joints are not required at the time of referral, ensuring inclusion of an important group of patients with new-onset inflammatory arthralgia into the resultant cohort. Such patients typically describe joint pain with morning stiffness, but have no clinically inflamed/swollen joints on examination. In this observational cohort study, we used the principles adopted by van der Helm-van Mil *et al*. [16] to construct a predictive algorithm for PIA amongst EA clinic attendees. We then asked whether the addition of the most predictive element(s) of a short, pragmatic MSUS screening protocol improved its predictive utility.

## Materials and methods

### Subjects and data collection

Consenting patients ≥16 years of age and presenting with new-onset arthralgia to the Newcastle EA clinic between September 2006 and November 2009 were included into the study. Detailed baseline demographic and clinical parameters were recorded during the patients' first EA clinic visit by an experienced specialist nurse, at which time routine blood tests included acute phase markers and autoantibodies. A "joint pattern score" (JPS) between 0 and 2.5 was also recorded for each patient, reflecting the localisation and distribution of symptomatic joints at presentation according to the system described by van der Helm-van Mil *et al*. [16]. An initial diagnosis was assigned to each patient by their consulting rheumatologist according to a "working diagnosis proforma" (Table 1) [15]. RA was diagnosed only where 1987 ACR classification criteria [17] were fulfilled; UA was defined as a "suspected inflammatory arthritis where RA remained a possibility, but where established classification criteria for any rheumatological condition remained unmet". This initial diagnosis was updated by the rheumatologist at each subsequent clinic visit for the duration of the study, which was greater than 12 months for all patients. A knowledge of outcome diagnoses was used to further categorise patients according to whether or not they developed a persistent inflammatory arthritis (PIA *versus *non-PIA). Hence, RA, psoriatic arthritis, enteropathic arthritis, ankylosing spondylitis, undifferentiated spondyloarthropathy, connective tissue disease and other inflammatory arthritides constituted PIA outcomes. A subset of individuals assigned the "self-limiting inflammatory/reactive arthritis" outcome, who had definite reactive arthritis warranting DMARD treatment, were also included in the PIA grouping. Remaining EA clinic attendees, diagnosed with self-limiting inflammatory arthritis, crystal pathologies, osteoarthritis or non-inflammatory arthralgia at follow-up, formed the non-PIA category. Enrolled patients consented to participate in the study, which received a favourable review by the Newcastle and North Tyneside Local Research Ethics Committee.

**Table 1:** Categorisation of working diagnoses

• RA			□
• UA			□

• Non-RA:	"Inflammatory"	Psoriatic arthritis	□
		
		Reactive/self-limiting inflammatory arthritis	□
		
		Ankylosing spondylitis*	□
		
		Enteropathic arthritis	□
		
		Undifferentiated spondyloarthritis (not RA)	□
		
		CTD	□
		
		Crystal	□
		
		Other	□
	
	"Non-inflammatory"	Osteoarthritis	□
		
		Noninflammatory arthralgia/other.	□

Categorisation of working diagnoses used amongst early arthritis patients at inception and follow-up during the course of this study. Consultant rheumatologists were asked to tick one box at each clinic visit, indicating the best description of their expert opinion of the diagnosis at a given time. See text. *Where modified New York criteria for the diagnosis of ankylosing spondylitis were not met, but the diagnosis was suspected in the context of seronegative inflammatory disease, consultants were asked to record a diagnosis of "undifferentiated spondyloarthritis".

### MSUS protocol

The same *Aplio*™ Diagnostic Ultrasound System (Toshiba Medical Systems Corporation, Tochigi-Ken, Japan) was employed for all MSUS assessments. This employed a 12 MHz probe, the screening protocol used in the study could be completed in approximately 10 minutes, and was performed in the EA clinic by one of three experienced MSUS practitioners. A total of 16 peripheral small joints were routinely evaluated: the second to fourth metacarpophalangeal (MCP) and proximal interphalangeal (PIP) joints bilaterally (dorsal and volar longitudinal planes, neutral and flexed position) and the first and second metatarsophalangeal (MTP) joints bilaterally (dorsal longitudinal plane only). Semiquantitative scores were assigned at each site for three "domains": grey-scale synovitis, power Doppler signal and bony erosion, according to consensus definitions [18,19]. For each domain at each hand joint, only the higher of the dorsal and volar scores was recorded. Each domain was scored on a 0 to 3 semi-quantitative scale, based on the system first suggested by Szudlarek *et al*. [20], but adopting the modification of Scheel *et al*. in respect of grey-scale synovitis, whereby effusion and synovial thickening were grouped to give a single, combined score [21]. In recognition that a degree of grey-scale synovitis at MTP1 may be physiological [13], a semi-quantitative score of 1 at this joint did not count towards the overall score for this domain. Once complete MSUS datasets had been obtained for 397 patients, five potentially useful dichotomous variables were identified with respect to diagnostic outcome. Broadly, these parameters represented either "synovitis load" within a defined number of joints (for example, the sum of semi-quantitative grey-scale synovitis scores of the 16 specified joints), or "proportionate joint involvement" (for example, the proportion of joints in which any power doppler signal was present); optimal cut-off values for each parameter were determined from the available datasets.

During preliminary work for the study, inter-observer agreement between assessors was determined. For example, 16 small hand/foot joints of 20 EA clinic patients (a total of 316 joints) were independently scanned by two practitioners (PNP and AGP), each being blinded to the recorded findings of the other at the time of documentation. Semi-quantitative scores of both grey-scale synovitis and power Doppler synovitis were dichotomised into the presence or absence of a score ≥1 for each joint, and resultant Kappa statistics (0.56 for grey-scale and 0.64 for power Doppler synovitis) demonstrated moderately good inter-observer agreement, which is comparable with that seen in other settings [22]. Comparable kappa statistics were obtained between other practitioners using the same method. Further preliminary work confirmed an excellent intra-observer reliability for small joint MSUS measurements. For example, anonomysed images from 18 patients, scored by AGP on separate occasions (two weeks apart) in random sequence for the presence/absence of grey-scale and power Doppler semi-quantitative scores ≥2, yielded kappa values of 0.85 and 0.91, respectively.

### Statistical analysis

Student's *t*-tests and Mann-Whitney U tests (parametric and non-parametric data, respectively), contingency table statistics (kappa tests and Pearson's chi-squared test, including Yates' continuity correction and effect size calculations for 2 × 2 tables), logistic regression analyses and the construction of receiver operator characteristic (ROC) curves were carried out using SPSS version 15 (SPSS Inc., Chicago, IL, USA).

For the primary logistic regression analyses of the complete Early Arthritis Clinic (EAC) cohort, a backward selection approach was used which rationally identified the most significant independent variables, with a *P*-value of 0.1 having been set as the removal criterion. Where variables were available in either continuous or categorical formats the choice of whether to enter a given variable in continuous or categorical format was made based on an iterative process, whereby the accuracies of derived models for discriminating EA-PIA *versus *EA-non-PIA were compared - only the derivation of the final model is outlined here. In the resultant regression models, the predicted probability of PIA was related to the covariates via the following prognostic index: B_1_x_1 _+ B_2_x_2_+... ...+B_n_x_n_, where × refers to a specific covariate, n is the total number of covariates in the model, and the regression coefficient (B) of each covariate indicates an estimate of the relative magnitude of its prognostic power. Using this prognostic index, it was possible to calculate the predicted probability of PIA developing for every EA patient. For ease of use, the values of regression coefficients (B) incorporated into the index were doubled and then rounded to the nearest 0.5 to provide a simplified prognostic index for clinical application, without substantially altering the prognostic utility of the tool. Where data were missing for individual subjects (within the "minimum dataset" constraints outlined above), median values from the final study cohort were imputed to enable multivariate analysis of the complete dataset. Variables requiring modification in this way (number, percentage of individuals) were: C-reactive protein (CRP) (5, 1%), erythrocyte sedimentation rate (ESR) (9, 2%), anti-citrullinated peptide autoantibody (ACPA) (9, 2%), and rheumatoid factor (RF) (3, 0.8%).

An entirely analogous approach was then used for the derivation of regression models for predicting an RA diagnostic outcome amongst the sub-cohort of patients classified as having PIA, and for predicting RA amongst those who presented with UA.

## Results

### EA patient cohort and univariate analysis of baseline characteristics

A total of 389 eligible patients were recruited between September 2006 and April 2009 inclusive, and were followed up for a minimum of 12 months (median 27; range 12 to 44 months); 10 had arthritis that remained undifferentiated at the end of the follow-up period, and were excluded from analysis. The diagnostic evolution of the remaining 379 patients is presented in Table 2. The baseline clinical and serological characteristics of patients in whom PIA did or did not develop are compared in Table 3, each of 12 variables being considered both categorically and continuously where possible; these were: age, sex, smoking status, symptom duration, tender joint count, swollen joint count, joint pattern score, early morning stiffness (EMS), ESR, CRP, RF and ACPA status. Five MSUS parameters for synovitis were identified as having potential discriminatory utility at baseline with respect to an outcome of PIA; namely, (i) "sum of scores (grey-scale domain) for total of 16 scanned joints," (ii) "sum of scores (grey-scale domain) for 6 scanned joints of worst-affected hand," (iii) "number out of 16 total scanned joints scoring ≥1 (grey-scale domain)", (iv) "sum of scores (power Doppler domain) for total of 16 scanned joints" and (v) "number out of 16 total scanned joints scoring >1 (power Doppler domain) (see also Methods). Findings in relation to these parameters, as well as the presence/absence of MSUS erosions were also compared (as dichotomous variables, according to pre-determined optimal cut-offs) between comparator groups (Table 3).

**Table 2:** Diagnostic evolution of EA patients

**Working diagnostic category (consulting rheumatologist's opinion)**.	**Number (%) assigned diagnosis (total = 379)**	
	Inception:	Close of follow-up: (median 28 months)
Undifferentiated arthritis	91 (24.0)	0 (-)
Rheumatoid arthritis	69 (18.2)	102 (26.9)
Psoriatic arthritis	24 (6.3)	25 (6.6)
Enteropathic arthritis	3 (0.8)	4 (1.1)
Reactive/self-limiting inflammatory arthritis	21 (5.5)	36 (9.5)
Ankylosing spondylitis	0 ( - )	2 (0.5)
Undifferentiated spondyloarthritis	7 (1.8)	9 (2.4)
Crystal arthritis (gout/pyrophosphate)	17 (4.5)	17 (4.5)
Connective tissue disease	5 (1.3)	7 (1.8)
Other inflammatory arthropathy	7 (1.8)	9 (2.4)
Osteoarthritis	66 (17.4)	78 (20.6)
Other non-inflammatory/arthralgia	69 (18.2)	90 (23.6)

Breakdown of inception and outcome diagnoses for 379 EA clinic patients.

**Table 3:** Univariate analysis of baseline clinical and MSUS parameters

Clinical/Laboratory Parameters:		Overall cohort (*n *= 379)	Outcome diagnostic category		
			
			Non-PIA (*n *= 217);	PIA (*n *= 162);	*P*-value†
**Age **(years)					
-cat; >50 (%)		51	49	51	0.533
-cont; mean (SD)		50.9 (36 to 66.)	48.5 (34.5 to 62.5)	53.8 (37.8 to 69.8)	0.057

**Sex**; F (%)		69	71	67	0.540

**Ever smoked? **(%)		54.1	100, 117	74, 88	0.999

**Sx durn **(weeks)					
-cat; ≥20 (%)		51	61	38	<0.001
-cont; median (IQR)		20 (10 to 34)	24 (12 to 52)	12 (8 to 24)	<0.001

**TJC **/72					
-cat; >6 (%)		51	48	54	0.299
-cont; median (IQR)		7 (2 to 14)	6 (2 to 14)	7.5 (3 to 15)	0.052

**SJC **/72					
-cat; ≥1 (%)		46	26	73	<0.001
-cont; median (IQR)		0 (0 to 3)	0 (0 to 1)	2 (0 to 6)	<0.001

**JPS**					
-cat; ≥2 (%)		66	56	78	<0.001
-cont; median (IQR)		2 (1.5 to 2)	2 (1.5 to 2)	2 (2 to 2.5)	<0.001

**EMS **(hours)					
-cat; ≥1 (%)		50	39	65	<0.001
-cont; median (IQR)		1 (0.2 to 2)	0.5 (0.05 to 1.5)	1 (0.5 to 2)	<0.001

**CRP **(g/l)					
-cat; <5,5 to 14,≥14 (%)		49,26,25	66, 23, 11	25, 30, 45	<0.001
-cont; median (IQR)		5 (<5 to 14)	<5 (<5 to 8)	11 (<5 to 32)	<0.001

**ESR **(mm/hr)					
-cat; >24 (%)		50	33	72	<0.001
-cont; median (IQR)		24 (13 to 50)	18 (9 to 13)	45 (24 to 67)	<0.001

**RF**; Pos (%)		Neg, Pos	193, 24	97, 65	<0.001

**ACPA**; Pos (%)		Neg, Pos	216, 1	107, 55	<0.001

**MSUS Parameters** ***Domain/Parameter*** ***Definition***					

***Grey-sale synovotis***	*∑ semi-quant. score/16 joints:* **≥2 **(%)	35.1	19.4	56.2	<0.001
	
	*∑ semi-quant score/6 joints (worst hand): ***≥2 **(%)	29.6	15	48.8	<0.001
	
	*Number of joints/16 scoring ≥1:* **≥3 **(%)	30.1	14.7	50.6	<0.001

***Power Doppler synovitis***	*∑ semi-quant. Score/16 joints:* **≥1 **(%)	29	15.7	46.9	<0.001
	
	*Number of joints/16 scoring ≥1:* **≥2 **(%)	16.9	7.4	29.6	<0.001

Baseline data for study cohort, and univariate analysis amongst patients with persistent inflammatory versus non-inflammatory diagnostic outcomes. Clinical and laboratory parameters are considered in both continuous (cont.) and categorical (cat.) formats. ACPA, anti-citrullinated peptide antibody; EMS, early morning stiffness; IQR, inter-quartile range; JPS, joint pattern score; RF, rheumatoid factor; SD, standard deviation, SxDur, symptom duration; T/SJC, tender/swollen joint count, (see Methods for derivation). Baseline MSUS data and corresponding univariate analysis (employing predetermined, optimal cut-off values for each dichotomous parameter) is also presented. ∑, sum of, semi-quant, semi-quantitative; PIA, persistent inflammatory arthritis. *Mann-Whitney U/Student's *t *tests for skewed/normally-distributed continuous data respectively; Pearson's χ^2 ^with Yates' continuity correction for dichotomous data.

### Predictive algorithm for PIA: no MSUS

**Figure 1:**
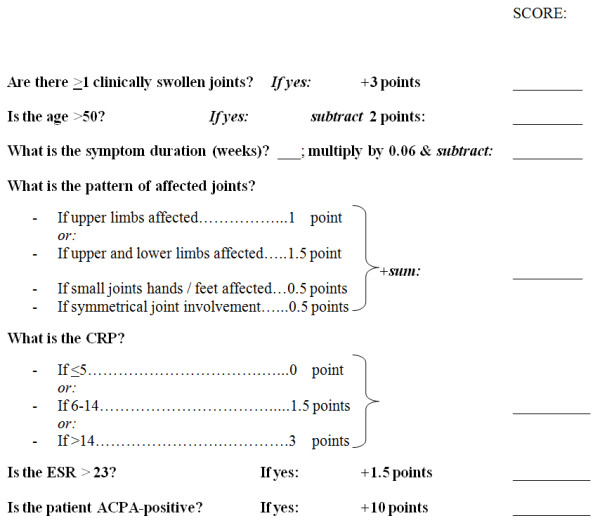
**Risk metric calculation tool (MSUS not required)**. Risk metric calculation tool for use in clinical practice, in which MSUS is not required.

**Figure 2:**
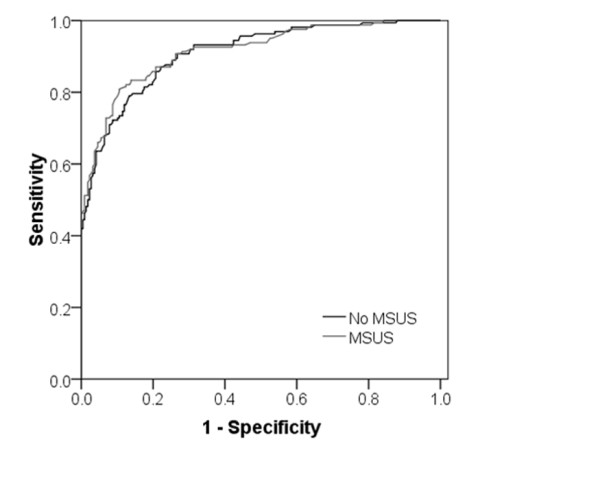
**ROC curve comparing discriminatory utility of predictive metrics for persistent inflammatory arthritis**. Receiver operator characteristic (ROC) curve illustrating the discriminatory utility of two risk metrics, derived excluding or incorporating musculoskeletal ultrasound (MSUS) parameters, with respect to an outcome of PIA ("No MSUS" and "MSUS" respectively; area under both curves 0.91; SEM 0.015; *P *< 0.001).

The 12 clinical/serological variables were entered into a backward stepwise logistic regression analysis, with PIA *versus *non-PIA outcome as the dependent variable (Table 4). Amongst 379 EA clinic patients, and after sequential removal of non-significant parameters, 7 variables were independently associated with PIA. The final model containing these predictors was significantly associated with PIA (χ^2 ^(7 degrees of freedom) = 240.4; *P *< 0.001), and explained between 47% (Cox and Snell R square) and 63% (Nargelkerke R squared) of the variance in diagnostic outcome [23]. A simple "risk metric" for clinical use could be calculated for each of the 379 individuals in the EA cohort, based in each case on the values of the seven independent predictive variables in the regression model [16] (Figure 1 and see also Methods). This risk metric was shown to have an excellent discriminatory ability in the current dataset through the construction of a ROC curve, the area under which was 0.91 (standard error of mean (SEM) = 0.015, *P *< 0.001; Figure 2).

**Table 4:** Predicting PIA in EAC patients: results of backward stepwise regression, excluding MSUS variables

Variable	Coding if categorical		B	SE	Wald	*P*-value	OR (95% CI)
**Age** (years)	<50:	0					
	≥50:	1	**-0.80**	0.32	6.27	**0.012**	0.45 (0.24 to 0.84
**Sx Durn** (weeks)	n/a		**-0.030**	0.01	12.64	**<0.001**	0.97 (0.96 to 0.99)
**SJC **/72	<1:	0					
	≥1:	1	**1.53**	0.31	23.94	**<0.001**	4.61 (2.50 to 8.43)
**JPS**	n/a		**0.42**	0.26	2.66	**0.100**	1.52 (0.92 to 2.52)
**CRP** (g/l)	<5:	0					
	5 to 14:	1	**0.77**	0.21	13.48	**<0.001**	2.16 (1.43 to 3.27)
	≥14:	2					
**ESR** (mm/h)	<24	0					
	≥24	1	**0.79**	0.34	5.33	**0.021**	2.21 (1.13 to 4.32)
**ACPA**	Neg:	0					
	Pos:	1	**4.89**	1.10	21.36	**<0.001**	132.4 (17 to 1052)
**Constant**			**-2.12**	0.52	16.63	**<0.001**	-

Results of backward stepwise logistic regression analysis to identify independent clinical (non-MSUS) predictors of PIA amongst EA clinic attendees. B values are regression coefficients. CI, confidence interval; OR, odds ratio; SE, standard error of B. Additional abbreviations as per Table 3. See text.

The incidence of PIA in our cohort was 162/379, or 0.42. Taking this to represent the prior probability of PIA amongst our EA cohort, and employing a single cut-off value for the prediction score, the positive and negative predictive values (PPV and NPV) of a score ≥4.0 were 0.83 in both cases (95% CIs 0.75 to 0.88 and 0.78 to 0.88, respectively), the positive and negative likelihood ratios (+LR and -LR) being 6.4 (4.41 to 9.25) and 0.27 (0.20 to 0.35), respectively. In the absence of external, independent validation, studies which rely on statistical modelling by logistic regression may lead to over-optimistic assessment of predictive utilities due to the phenomenon of "over-fitting" [23]. By employing a stringent 10-fold random sub-sampling cross-validation to account for this possibility [24], our model's PPV with respect to PIA reduced to 0.72 (0.68 to 0.76), but the NPV was maintained at 0.85 (0.83 to 0.88) (+LR and -LR corrected to 3.56 (3.10 to 4.08) and 0.22 (0.18 to 0.26), respectively).

### Predictive algorithm for PIA is not improved by incorporation of MSUS parameter(s)

**Figure 3:**
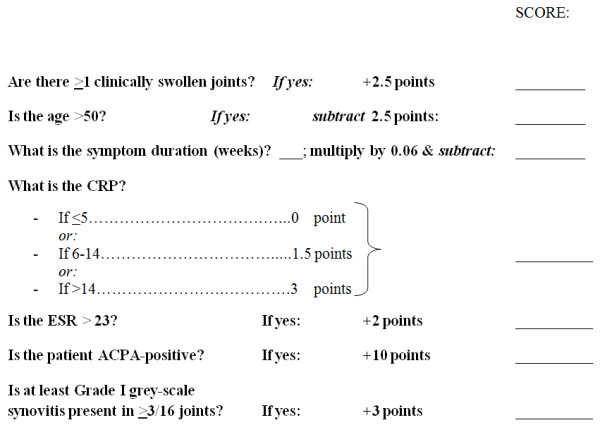
**Risk metric calculation tool (includes MSUS parameter)**. Revised risk metric calculation tool for use in clinical practice, derived from baseline variables that included musculoskeletal ultrasound (MSUS) parameters; an assessment of grey-scale synovitis is a required component of the resultant risk metric.

Backward stepwise logistic regression analysis was repeated, with the addition of the five discriminatory MSUS parameters as independent variables alongside the seven clinical/serological variables previously identified (and listed in Table 4). The results of this revised multivariate analysis are presented in Table 5, which identifies a predictive model very similar to that previously derived, comprising seven independent predictors of PIA, but with "joint pattern score" being replaced by the MSUS parameter, " ≥3/16 specified joints with any grey-scale synovitis". This model was again significantly associated with outcome (χ^2 ^(7 degrees of freedom) = 255.8; *P *< 0.001), explaining between 49% (Cox and Snell R square) and 66% (Nargelkerke R squared) of the variance in diagnostic outcome [23]. The simplified risk metric for clinical use was then revised to incorporate the new MSUS parameter in place of joint pattern score (Figure 3). Based on the ROC curves constructed from metrics that did or did not include MSUS parameter(s), the diagnostic utility of the new metric (which required MSUS) was seen to be equivalent to, but not superior to, that derived from more readily obtainable clinical and serological parameters alone (area under both curves 0.91; SEM 0.015; *P *< 0.001) (Figure 2).

**Table 5:** Predicting PIA in eac patients: results of backward stepwise regression, incorporating MSUS variables

Variable	Coding if categorical		B	SE	Wald	*P*-value	OR (95% CI)
**Age** (years)	<50:	0					
	≥50:	1	**-1.24**	0.36	11.81	**0.001**	0.29 (0.14 to 0.59)
**Sx Durn** (weeks)	n/a		**-0.03**	0.01	11.182	**0.001**	0.97 (0.96 to 0.99)
**SJC **/72	<1:	0					
	≥1:	1	**1.42**	0.32	19.21	**<0.001**	4.14 (2.19 to 7.80)
**CRP** (g/l)	<5:	0					
	5 to 14:	1	**0.64**	0.22	8.43	**0.004**	1.89 (1.23 to 2.92)
	≥14:	2					
**ACPA**	Neg:	0					
	Pos:	1	**4.97**	1.07	21.74	**<0.001**	143.7(18 to 1060)
**ESR** (mm/h)	24:	0					
	≥24:	1	**1.00**	0.36	7.74	**0.005**	2.72 (1.34 to 5.51)
**Presence of *Grade I *grey-scale synovitis in ≥3/16 joints**	No:	0					
	Yes:	1	**1.6**	0.34	17.22	**<0.001**	4.91 (2.32 to 10.4)
							
**Constant**			**-1.62**	0.34	22.68	**<0.001**	-

Results of logistic regression analysis of 5 MSUS paramteres in addition to 7 previously identified independent predictors for PIA amongst 379 patients of EAC cohort. B values are regression coefficients. CI, confidence interval; OR, odds ratio; SE, standard error of B. Additional abbreviations as per Table 3. See text.

### A predictive algorithm for RA amongst the PIA sub-cohort is not improved by the incorporation of MSUS parameter(s)

**Figure 4:**
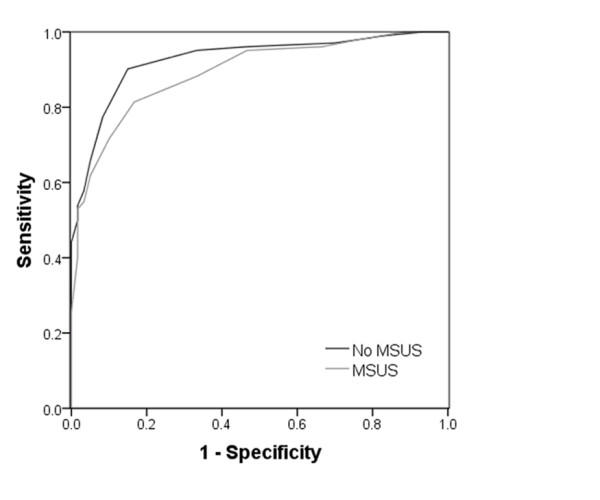
**ROC curve comparing discriminatory utility of predictive metrics for RA amongst PIA sub-cohort**. Receiver operator characteristic (ROC) curve illustrating the discriminatory utility of two risk metrics derived amongst the PIA sub-cohort alone, excluding or incorporating musculoskeletal ultrasound (MSUS) parameters, with respect to an outcome of rheumatoid arthritis (RA) ("No MSUS" and "MSUS" respectively). Area under curves: 0.93 (No MSUS; SEM 0.021; *P *< 0.001), 0.89 (MSUS; SEM 0.025; *P *< 001).

Using the principals applied to the early arthritis cohort as a whole, described above, simplified risk metrics were developed to discriminate between PIA patients diagnosed with RA *versus *alternate inflammatory arthritides, exploring the value added for such purposes by the incorporation of MSUS. Hence, excluding MSUS parameters, backward stepwise regression identified five variables independently associated with an outcome of RA in this sub-cohort (*n *= 162); namely age, swollen joint count, joint pattern score, ESR and ACPA status (Table 6). By additionally entering five discriminatory MSUS parameters into the regression analysis a similar predictive model was derived, comprising five independent variables, which differed only in that a dichotomous power Doppler semi-quantitative score (total ≥1 for total of 16 scanned joints) replaced swollen joint count (SJC) ≥1 (Table 7). However, the respective discriminatory ability of simplified risk metrics derived from the two predictive models were comparable, suggesting that MSUS did not add value to clinical and serological parameters alone in identifying PIA patients who develop RA (Figure 4). Further additive roles for MSUS in predicting clinically relevant outcomes in early arthrtis patients were explored. For example, using similar approaches, we were unable to demostrate its enhanced ability to predict PIA in a sub-cohort of EA clinic attendees who present without clinical evidence of swollen/inflamed joints, or to predict progression to RA amongst those individuals who present with UA (*n *= 204 and *n *= 91, respectively; data not shown).

**Table 6:** Predicting RA in PIA patients: results of backward stepwise regression, excluding MSUS variables

Variable	Coding if categorical		B	SE	Wald	*P*-value	OR (95% CI)
**Age** (years)	<50:	0					
	≥50:	1	**1.52**	0.49	9.52	**0.002**	4.58 (1.74 to 12)
**SJC **/72	<1:	0					
	≥1:	1	**1.26**	0.55	5.21	**0.023**	3.52 (1.19 to 10)
**JPS**	n/a		**1.38**	0.50	7.67	**0.006**	3.99(1.50 to 11)
**ESR** (mm/h)	<24	0					
	≥24	1	**1.14**	0.53	4.63	**0.031**	3.13 (1.11 to 8.85)
**ACPA**	Neg:	0					
	Pos:	1	**4.30**	0.88	24.17	**<0.001**	74 (13 to 409)
**Constant**			**-5.61**	1.26	20.0	**<0.001**	-

Results of backward stepwise logistic regression analysis to identify independent clinical (non-MSUS) predictors of RA amongst PIA sub-cohort. B values are regression coefficients. CI, confidence interval; OR, odds ratio; SE, standard error of B. Additional abbreviations as per Table 3. See text.

**Table 7:** Predicting RA in PIA patients: results of backward stepwise regression, incorporating MSUS variables

Variable	Coding if categorical		B	SE	Wald	*P*-value	OR (95% CI)
**Age** (years)	<50:	0					
	≥50:	1	**1.26**	0.51	6.23	**0.013**	3.59 (1.32 to 9.81)
**Presence of *GradeIP*ower Doppler synovitis in ≥1/16 joints**	No:	0					
	Yes:	1	**1.70**	0.52	10.72	**0.001**	5.46 (1.98 to 15.1)
**JPS**	n/a		**1.39**	0.54	6.60	**0.010**	4.02(1.39 to 11.6)
**ESR** (mm/h)	24:	0					
	≥24:	1	**1.17**	0.56	4.48	**0.034**	3.24 (1.09 to 9.60)
**ACPA**	Neg:	0					
	Pos:	1	**4.06**	0.82	24.74	**<0.001**	58.1(12 to 288)
**Constant**			**-5.38**	1.28	17.64	**<0.001**	-

Results of logistic regression analysis of maximally discriminatory MSUS paramters in addition to previously identified independent predictors for UA amongst 162 patients of PIA sub-cohort. B values are regression coefficients. CI, confidence interval; OR, odds ratio; SE, standard error of B. Additional abbreviations as per Table 3. See text.

## Discussion

We present a predictive algorithm, developed in a "true-to-life" EA clinic setting in the UK (Figure 1), which may be used to estimate the probability that an individual EA clinic attendee will develop a PIA. Predicting persistent inflammatory disease amongst EA clinic attendees, rather than mere fulfilment of classification criteria for RA (based on the 1987 ACR classification criteria for the disease), sets our study's objectives apart from those of van der Helm-van Mil *et al*. [7,16], but our approach bears similarities with those of others. In the well-known example of Visser *et al*., an 8-point scoring system for use amongst EA patients was developed (maximum score 13) that permitted the probablility of PIA to be calculated for an individual patient [11]. Unsurprisingly, the component parameters identified in that analysis overlapped with those of the current study (Figure 1), particularly emphasising the predictive importance of symptom duration, distribution of involved joints and ACPA status for PIA. Unlike the cohort studied by Visser *et al*., however, it is important to note that ours included some patients with no objectively swollen joints at baseline; this was in order to capture the clinically important "inflammatory arthralgia" group. This probably explains the inclusion of swollen joint count and CRP parameters in our own (but not in Visser *et al*.'s) predictive model.

Our study addressed a highly relevant clinical question: whether MSUS, considered alongside more readily obtainable clinical and laboratory measurements, helps to predict PIA amongst unselected EAC patients referred from primary care with recent-onset joint pain. The comprehensiveness of any MSUS screening protocol used for this purpose needs to be balanced by the feasibility of its use as part of a time-constrained, routine clinical assessment, and we developed a protocol that could in most cases be completed within 10 minutes, focussing on small peripheral joints. In our hands, MSUS parameters provided no additional discriminatory value under these circumstances (Figure 2). The independent contribution of ACPA status to predictive models for PIA was remarkable, being reflected in the magnitude of its associated regression coefficient (4.89; Table 4), implying that the adoption of autoantibody testing as a diagnostic tool in this setting provides superior discriminatory utility over the MSUS screening parameters presented here. With 57% of EAC patients having non-inflammatory outcomes, it may be argued that the discriminatory utility of MSUS would be better defined when discriminating patients with an outcome of classifiable RA amongst the sub-cohort with PIA, but this was not found to be the case in our study (Figure 4). Neither were we able to demonstrate an additive predictive utility of MSUS when predicting progression of (i) arthralgia (in the absence of clinically evident synovitis) to PIA, or (ii) UA to RA - although our study lacked power to exclude either of these comprehensively. It is nonetheless noteworthy that the backward selection procedure employed during regression analyses suggested that the independent association amongst PIA patients of RA outcome with the presence of one or more clinically swollen joints was less strong than it was with the presence of any power Doppler signal in the 16 screened peripheral joints (compare respective odds ratios, Tables 6 and 7). The quantitative lack of additive discrimination provided by MSUS in these settings does not, therefore, negate the value of this imaging modality as an extension of the clinical examination in the evaluation of early arthritis.

Our observational study has a number of limitations, primarily reflecting the opportunistic manner in which data were collected in a real-time, real-life and busy EA clinic. Data published by others during the course of our study have suggested that the wrists and (albeit in the longitudinal evaluation of established disease) the fifth MTPJs may have particular value as sensitive markers of inflammatory arthritis [13,25], but these joints were not included in our routine screening algorithm. Conversely the first MTPJ frequently contains an effusion of equivocal significance and is the least informative MTPJ [13]. Although we discounted a semi-quantitative greyscale score of 1 at this site (see Methods), our inclusion of the first MTPJ may, therefore, have influenced our findings, and our routine screening protocol might have yielded superior discriminatory value had it been informed by the detailed pilot work of Filer *et al*. [13]. Further work is required to define an optimal short MSUS screening protocol for use in the early arthritis setting, and, in particular, the extent to which it should include extra-articular sites, for example, to determine the presence of tenosynovitis or enthesitis. Finally, the definition of PIA was pragmatic, being based upon outcome diagnoses at follow-up, and the classification of each diagnosis for this purpose was not intuitive in all cases. For example, it was decided to define patients with crystal arthropathy (gout and calcium pyrophosphate deposition) as non-PIA, since such patients are not considered candidates for DMARDs, and their appropriate management in this cohort ensured that inflammatory manifestations were, for the most part, self-limiting. Their number was relatively small (*n *= 17), and classifying them alternatively as PIA did not materially alter the overall outcome of the analysis.

Notwithstanding the above, we set out to address whether MSUS parameters added to more readily obtainable clinical and laboratory data in the generation of predictive models in the EAC. In this regard, our large EAC cohort adds a substantial dataset to inform current knowledge and debate.

## Conclusions

A "risk metric" using baseline clinical parameters predicts early arthritis patients who develop PIA. The incorporation of MSUS parameters does not add discriminatory value to the risk metric. Further studies will determine the role of MSUS in the diagnosis of early arthritis.

## Abbreviations

ACPA: anti-citrullinated peptide antibodies; ACR: American College of Rheumatolgy; CRP: C-reactive protein; DMARD: disease modifying anti-rheumatic drug; EA: early arthritis; ESR: erythrocyte sedimentation rate; JPS: joint pattern score (defined in text); LR: likelihood ratio; MCP(J): metacarpo-phalangeal (joint); MSUS: musculoskeletal ultrasound; MTP(J): metatarso-phalangeal (joint); PIA: persistent inflammatory arthritis; PIP(J): proximal interphalageal (joint); RA: rheumatoid arthritis; ROC: receiver operator characteristic; SEM: standard error of the mean; SJC: swollen joint count; TJC: tender joint count; UA: undifferentiated arthritis.

## Competing interests

The authors declare that they have no competing interests.

## Authors' contributions

AGP designed the study, carried out MSUS assessments, analysed the data and wrote the manuscript. ARL carried out MSUS assessments and contributed to manuscript re-drafts. GW contributed to the study protocol, carried out clinical assessments of patients, and helped analyse clinical data. PNP helped design the MSUS assessment protocol and carried out or supervised all MSUS assessments. JDI supervised the study's design, analysis, and manuscript preparation. All authors read and approved the final manuscript.
